# A Window Into Mental Health: Developing and Pilot-Testing a Mental Health Promotion Intervention for Mexican Immigrants Through the *Ventanilla de Salud* Program

**DOI:** 10.3389/fpubh.2022.877465

**Published:** 2022-04-15

**Authors:** Delia Lilian Martínez Rodríguez, Tonatiuh González Vázquez, Margarita Márquez Serrano, Mary de Groot, Alicia Fernandez, Ines Gonzalez Casanova

**Affiliations:** ^1^School of Public Health of Mexico, National Institute of Public Health, Cuernavaca, Mexico; ^2^Oaxaca Health Services, Oaxaca, Mexico; ^3^Center for Health Systems Research, National Institute of Public Health, Cuernavaca, Mexico; ^4^Indiana University School of Medicine, Indianapolis, IN, United States; ^5^Department of Medicine, San Francisco School of Medicine, University of California, San Francisco, San Francisco, CA, United States; ^6^Department of Applied Health Science, Indiana University Bloomington School of Public Health, Bloomington, IN, United States; ^7^Hubert Department of Global Health, School of Public Health, Emory University, Atlanta, GA, United States

**Keywords:** mental health, health promotion, Mexican immigrants in the United States, community based mental health, protective factors, coping strategies

## Abstract

**Background:**

Mexican immigrants in the United States face mental health challenges, disparities, and limited access to healthcare; however, mental health promotion efforts specifically targeting this population have been insufficient. The objective of this study was to develop and test a mental health promotion intervention based on protective mental health factors and coping strategies for Mexican immigrants recruited through a free, consulate-based program in Atlanta.

**Material and Methods:**

Working with the Ventanilla de Salud program, we conducted a longitudinal study in three phases: formative research and design, pre-intervention assessment and post-implementation evaluation. The intervention was designed based on the health promotion model and interviews with stakeholders. Qualitative information was collected by semi-structured interviews with participants before and after the intervention. Quantitative outcomes were knowledge about protective factors and coping mechanisms, and psychosocial distress. Differences were assessed using the Wilcoxon non-parametrical test. Intent-to-treat analysis was conducted with all participants who signed the informed consent (carrying last observation forward), and a complete case analysis was conducted with those who attended at least 70% of the sessions and completed the post- implementation evaluation.

**Results:**

Twenty-five participants were enrolled in the intervention. Mean age was 38 years, and the majority were women. Only nine participants attended at least 70% of the sessions and completed the final evaluation. Men, those who did not complete high school, and workers in service or construction jobs were more likely to drop out. Knowledge about protective factors [pre- vs. post-intervention median (inter-quartile range) = 111 (100, 120) vs. 115 (100, 124)] and coping mechanisms [96 (85, 104) vs. 99 (90, 110)], as well as psychosocial distress [3 (2, 3) vs. 2 (2, 3)] improved after the intervention in both intent-to treat and complete case analyses (*p* < 0.05). Qualitative results also support improvements in targeted protective factors.

**Discussion:**

The intervention was successful in improving psychological distress among Mexican immigrants. These results support the implementation of evidence-based mental health promotion interventions among Mexican immigrants via free and familiar programs. A limitation was the high attrition; future studies should explore approaches to improve retention in this population.

## Introduction

Mexicans are the second largest migrant group in the world and the largest immigrant group in the United States, where there were ~12.3 million in 2019 (1). This population faces migration-related mental health challenges, socioeconomic disparities, and limited access to healthcare (2). It is estimated that, in 2019, 5.6 million Mexican immigrants in the US were undocumented, 20% lived in poverty and ~38% did not have access to health insurance (3). This makes linking Mexican immigrants to preventive services or healthcare very challenging, and often results in an accelerated deterioration of the health and wellbeing of this population, which translates into human and economic losses.

Latin American immigrants, including Mexicans, are at high risk of psychological distress and mental illness. A study of mental health among recently arrived Latino immigrants found a prevalence of depression of 26%—three times that of the US population (4). Similarly, migration-related loss and distress were present in all participants in a recent study of undocumented immigrants from Mexico (5). A political climate in the US that emphasized prosecution of undocumented immigrants, fear of being deported, language barriers, and socioeconomic challenges also has impacted the mental health of recent Mexican immigrants. Depression, anxiety, stress, addictions, gender violence, acculturative stress, and Immigrant Syndrome of Chronic and Multiple Stress (also known as Ulises Syndrome) have all been associated with the process of migration (6–8).

Interventions that support the development of coping strategies have been successful in improving mental health and preventing depression and anxiety symptoms in minoritized populations (9, 10). Coping strategies have been defined as group or individual decisions that support adaptation, managing new situations, problem solving, and emotional regulation when dealing with loss or stress. These strategies are the foundations for protective factors including adaptability, social support, and healthy habits (11). Mental health promotion interventions based on coping strategies could be especially important for Mexican immigrants; however, reaching this population can be challenging, and the lack of culturally tailored interventions has made addressing mental health risk factors in this group particularly difficult. Perhaps because of this, there are limited examples in the literature of mental health promotion interventions for Mexican immigrants in the United States (12–15). Most of these efforts have focused on screening and referrals rather than on education or training in mental health coping skills. In this study, we took advantage of a Ventanilla de Salud (VDS) which allowed us to reach Mexican immigrants through a free and familiar program, and design to design and test an evidence-based intervention with the potential to be scaled up through the VDS program.

The VDS program is a health promotion initiative offered through the fifty General Consulates of Mexico in the United States. A very large program, estimated to interact with over one million immigrants per year (16), it plays an important role providing preventive services and linkage to healthcare for Mexican immigrants and could provide an opportunity to address the gap in mental health promotion services among immigrants. Accordingly, as part of a thesis project (17), we developed a mental health promotion intervention based on coping strategies and protective factors and pilot-tested it among Mexican immigrants recruited through the VDS program in Atlanta, which provides preventive services and health education to Mexican immigrants in the Southeast of the United States.

## Methods

### Overview and Theoretical Framework

We conducted a longitudinal study in three phases: formative research and design, pre-intervention assessment and post implementation evaluation. We used a mixed methods approach based on the health promotion model that conceptualizes health as a positive state and aims to improve individuals' wellbeing. This model focuses on individual experiences, behavior-related cognitions and affect, and behavioral outcomes (18). Quantitative and qualitative information was collected in these domains.

For the development of the intervention, we followed the World Health Organization's recommendations for mental health promotion that identifies protective and risk factors (19). Protective factors of mental health refer to conditions that improve people's resilience to risk factors and disorders. They have been defined as those factors that modify, ameliorate, or alter a person's response to some environmental hazard that predisposes to a maladaptive outcome. Risk factors are associated with an increased probability of onset, greater severity and longer duration of major mental health problems (20, 21). In this case, we considered social support networks, adaptability, positive mental health habits, and coping mechanisms, including emotional regulation and stress management when faced with problems, as target protective factors for the intervention.

The educational sessions of the intervention were based on emotional education, which has been previously applied with adults for health purposes (22, 23) and is defined as an educational process, continuous and permanent, whose objective is to promote emotional development as a complement to cognitive development, for the development of a holistic personality. To do this, emotional education promotes the development of knowledge and skills about emotions in order to enable the individual to better face the challenges that arise in daily life (24). Objectives of emotional education are to acquire a better knowledge of one's own emotions, identify the emotions of others, develop the ability to regulate one's own emotions, prevent the harmful effects of negative emotions, develop the ability to generate positive emotions and the ability to positively relate emotionally with others (25).

### Setting

The General Consulate of Mexico in Atlanta serves primarily Mexicans living in the states of Georgia, Alabama, and Tennessee. Within this consulate, the VDS Atlanta offers preventive services and health promotion through education, glucose, weight and hypertension screenings, and referrals to community clinics and low-cost services. In 2019, the VDS Atlanta was funded through a partnership between the Mexican Government and Emory University's School of Public Health and provided services to ~30,000 people.

### Formative Research

The formative research consisted in non-participatory ethnographic observation of the functioning, practices and services provided at the VDS Atlanta, three semi-structured interviews with VDS personnel, and a group interview with three VDS clients. The interviews were conducted by the team psychologist (DLMR) and lasted ~30 min. The topics covered in the interviews were activities and programs conducted by the VDS, barriers to mental health care and prevention, and mental health challenges faced by Mexicans living in the Southeast of the US. Information was summarized into a report highlighting recommendations and potential challenges to implement a mental health intervention. The content of the intervention was adjusted based on results from this phase.

### Design and Implementation

**Figure 1:**
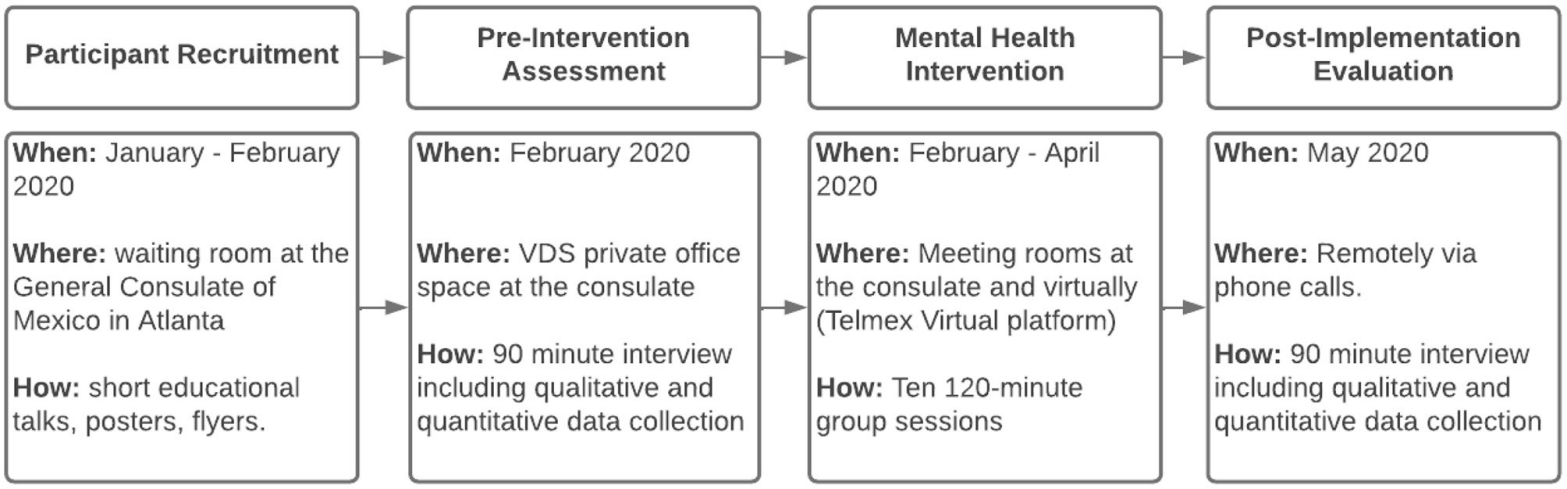
Intervention flowchart.

The design and implementation of the mental health intervention at the General Consulate of Mexico in Atlanta is summarized in Figure 1 and a detailed description is presented next.

### Participant Recruitment

Participant recruitment was conducted in person at the General Consulate of Mexico in Atlanta between December 2019 and February 2020. The team psychologist (LM) conducted 5–15 min health education talks for Mexicans in the waiting room of the consulate while their documents were being processed and invited them to participate in the intervention; additionally, posters and promotional materials were posted at the consulate, and an invitation to participate in the intervention was posted on the VDS Atlanta Facebook page. Interested participants received a screening and were enrolled in the study if they met the inclusion and exclusion criteria and provided written informed consent to participate in the study. The study was a project to obtain a master's degree in public health (17) approved by Ethics Board of the Mexican National Institute of Public Health (534-R71) and by Emory University Institutional Review Board (IRB00114514).

Inclusion criteria included: age 18 years or older, expressing need or interest in improving their mental health during their visit to the VDS, having an electronic device with internet connection, and being able to read and write. Exclusion criteria included: presence of a severe mental illness that required acute clinical care or having a visual, auditive, or cognitive impairment that impeded their participation in the sessions or in the evaluation.

### Intervention Design

This pre-post single arm intervention consisted of ten 120-min group sessions that covered protective factors (social networks, healthy mental health habits, adaptability) and coping strategies (emotional regulation, stress management, and tools to deal with anxiety).

The intervention was conducted between February and May 2020. For the first three sessions, in-person and online options were provided; the remaining sessions were online only. The online component was planned in response to the formative research phase based on concerns related to participants traveling to the consulate on a weekly basis, and on the location of the team psychologist who is based in Mexico. The intervention period also coincided with the COVID-19 pandemic, limiting travel. The online sessions were conducted synchronically through the Virtual platform; they were also recorded and posted on the closed Facebook group that was created for the intervention participants. The closed Facebook group was used as an additional tool to reinforce the session content and was used by the team psychologist to provide instructions and additional material on a weekly basis. Links to the weekly synchronous meetings and pre-post evaluation surveys were posted on this page.

Participants also were given the opportunity of up to two one-on-one meetings with the team psychologist after sessions 5 and 8. The objective of these sessions was to reinforce the material that was being covered during the sessions, to discuss individual progress and challenges, and to address any concerns or issues being faced by the participants. These sessions lasted 45–60 min. Throughout the intervention, the psychologist observed participants to see if there was additional need of emotional support through community referrals.

### Evaluation

The evaluation included a quantitative and a qualitative component before and after the intervention (pre-intervention assessment and post-implementation evaluation), and a process evaluation.

The pre- intervention assessment was conducted in person at the General Consulate of Mexico in Atlanta. It had an average duration of 90 min including the qualitative and quantitative components. The process evaluation was conducted during the third and seventh session to assess the method, materials, logistics, activities, and tools; it had the goal of identifying salient issues and improving the remaining sessions. The post-implementation survey and interview evaluation was conducted remotely a month after the last session.

### Quantitative Data Collection

#### Knowledge

This questionnaire measured knowledge about mental health protective factors, based on the Mental Health and Coping Skills of College Students, and knowledge about coping skills based on the Nursing professional coping attitudes questionnaire, which was used in Hispanic populations (26), and was adapted and pilot tested for this study with returning migrants in Mexico These questionnaires have 38 and 30 questions, respectively, follow a Likert scale, and took ~20 min to complete. The mental health protective factors knowledge questionnaire can be categorized to assess the level of knowledge into “very low” (1–47), “low” (48–95), “medium” (96–142) and “high” (143–190), it has high validity (*p* < 0.01) and reliability (α = 0.93) (26). The coping skills questionnaire can be categorized into “deficient” (1–37), “low” (38–75), “medium” (76–112), and “high” (113–150) and also had high validity (*p* = 0.02) and reliability (α = 0.70) (27).

#### Psychological Distress

We used the Kessler Psychological Distress Scale (20), a 10-item questionnaire designed to provide a global measure of distress based on questions about anxiety and depressive symptoms. It uses a Likert Scale and provides an assessment of psychosocial distress: “low” (10–15), “moderate” (16–21), “high” (22–29) and “very high” (≥30) (28, 29).

### Qualitative Data Collection

#### Semi-structured Interviews

Semi-structured interviews were conducted during the pre and the post evaluation. The pre- intervention interview was conducted in person in the Ventanilla de Salud private office space at the consulate and lasted ~45–60 min. The topics covered included sociodemographic information, family and individual mental health history, current mental health, effects of migration on mental health, protective factors and coping mechanisms. People with severe mental illness were excluded from the study and referred to local community resources.

The post- interviews were conducted remotely a month after the last session, they also had a duration of 45–60 min and included the following themes: current mental health status, protective factors and coping skills.

### Data Analysis

#### Quantitative

Data collected using the questionnaires were entered into an Excel (v16.3) dataset. Likert scales were converted into numerical values going from 1 to 5. To assess changes in pre- and post-values we used the Wilcoxon non-parametric test, this was selected due to the small sample size and because the data did not meet the normality assumption. Following analysis recommendations for pre-post designs (30), the intent-to-treat analysis included all participants with pre-intervention information and complete case analysis was restricted to participants who attended at least 70% of the sessions and had post-implementation information. The process evaluation data were entered into a Google Form and then descriptive statistics were used to analyze them. All quantitative analyses were conducted using STATA v.15.

#### Qualitative

Interview data were transcribed into a Microsoft Word (v16.3) document and content matrices were created manually. These were analyzed using deductive codes based on the theoretical framework and the themes that were pre-specified during the development of the interview guides. This was further complemented using inductive coding, incorporating themes that had not been considered a priori.

Quantitative and qualitative data was triangulated for the final intervention evaluation report.

## Results

### Baseline Characteristics

**Figure 2:**
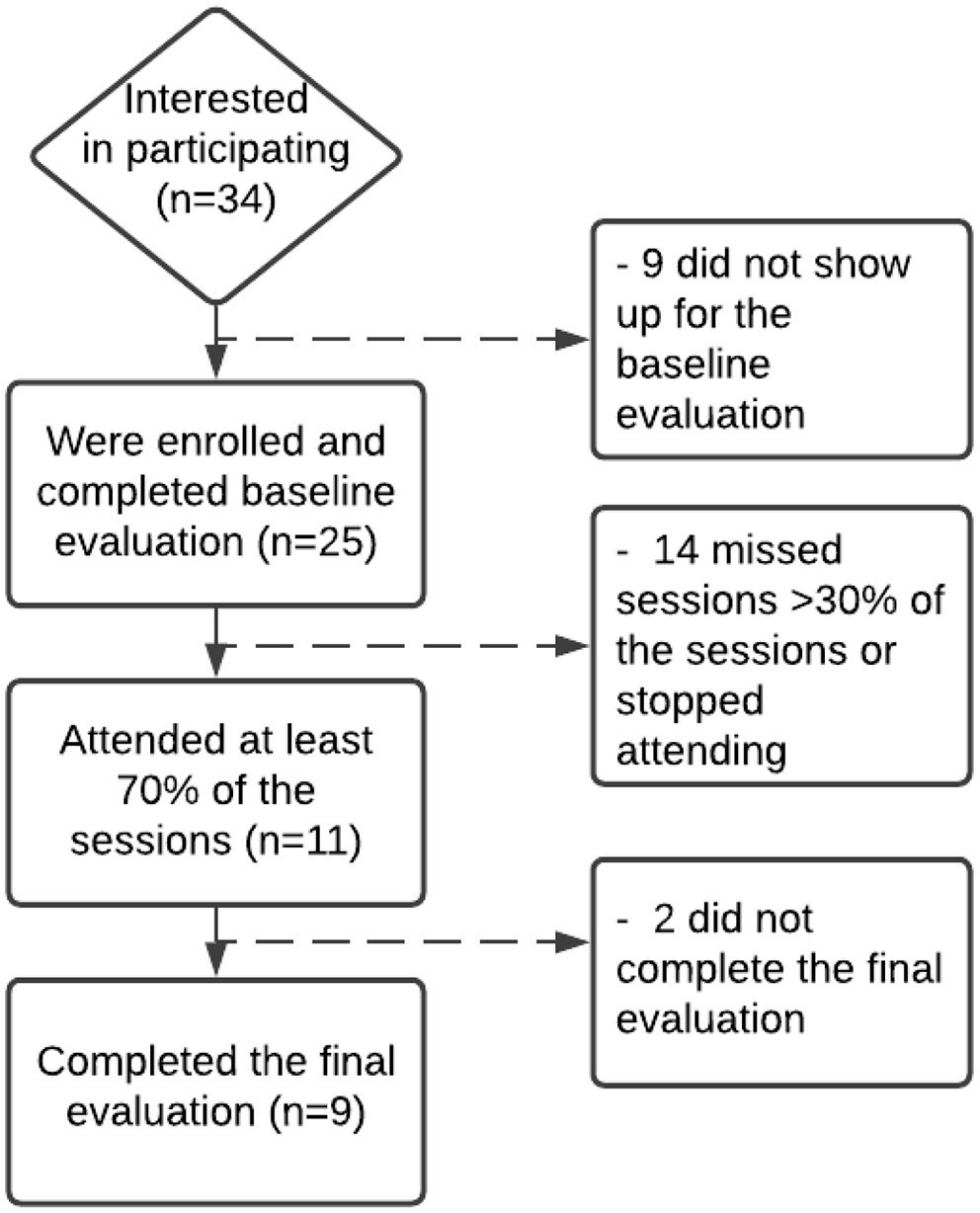
Participant flowchart.

Thirty-four participants expressed interest in participating, met the inclusion criteria, and were invited to the complete the pre-evaluation and participate in the sessions. Twenty-five participants signed the informed consent, completed the pre-evaluation, and were added to the Facebook group. Nine attended at least 70% of the sessions and completed the post-evaluation (Figure 2). The average attendance per session was of eleven people, with a minimum attendance of eight and a maximum of thirteen. Most participants who were lost to follow up did not show up to any session. The main reported reasons for not showing up were technical challenges using the platform (*n* = 6), time constraints (*n* = 4), unexpected circumstances such as change of job (*n* = 3), fear of legal consequences as an undocumented immigrant (*n* = 2), and shame of speaking in front of other participants (*n* = 1).

### Sociodemographic Characteristics of Study Participants

Participants were on average 39.8 (SD = 9.5) years and the majority (89%) were women. In general, participants that were included in the intervention were similar to those who were excluded from the pre-post evaluation due to missing sessions or not completing the post-evaluation. The main differences between these two groups were that workers in construction, cleaning, or cooking, and participants located outside of Georgia were more likely to not participate in 70% of sessions (Table 1).

**Table 1:** Sociodemographic characteristics of participants included (*n* = 9) and excluded (*n* = 16) from the pre-post evaluation.

**Characteristics [mean ±SD or *n* (%)]**	**Lost to follow-up^a^ (*n* = *16*)**	**Included in post-implementation evaluation** **(*n* = *9)***
Age (years)	37.8 ± 6.5	39.8 ± 9.5
Female	10 (62)	8 (89)
**Migratory origin**		
Traditional migratory region (Zacatecas, San Luis Potosí, Oaxaca, Michoacán, Guerrero)	9 (56)	5 (56)
Central Region (Mexico City, Mexico State, Hidalgo, Jalisco)	6 (38)	3 (33)
North region (Tamaulipas, Durango)	1 (6)	1 (11)
**Married or living with partner**		
Married or living with partner	10 (63)	7 (78)
Single/divorced/ widowed	6 (37)	2 (32)
**Number of children living in the US**		
0	2 (12)	2 (22)
1–3	11 (69)	6 (67)
4–6	3 (19)	1 (11)
**Schooling**		
Elementary school	3 (19)	0
Some high school	12 (75)	6 (37)
High school degree	0	1 (11)
Bachelor degree	1 (6)	2 (22)
**Ocupación**		
House wife	3 (19)	4 (44)
Assistants or supervisors (health, legal, bank)		4 (44)
Construction	6 (37)	
Cooks	3 (19)	1 (11)
Cleaning	4 (25)	
**Migratory status**		
US citizen		2 (22)
Permanent resident	2 (12)	1 (11)
DACA		2 (22)
Undocumented	11 (69)	4 (44)
Work permit	2 (12)	
Refugee	1 (6)	
**State**		
Georgia	6 (37)	9 (100)
North Carolina	3 (19)	
South Carolina	4 (25)	
Tennessee	1 (6)	
Time in the US (years)	13 ± 8.8	19 ± 3.9
Have health insurance	3 (19)	2 (22)

a *Participants who attended <70% of the sessions or did not complete the post-evaluation were considered lost to follow-up*.

### Group Characteristics From Qualitative Pre-intervention Evaluation (*n* = 25)

Five topics related to the characteristics of the group were identified in the semi-structured interviews implemented during the pre-evaluation: reasons for migration, immigration experience, physical and mental health, risk factors for mental health, and challenges accessing mental health services.

#### Reasons for Migration

Participants mentioned being encouraged by others as important reasons to migrate to the US. Some had been brought by their parents when they were still underage. Women mentioned traditional gender roles as the reason to come to the US following their husbands or sons. Economic factors also influenced this decision, some participants mentioned that their parents or partners suggested that they migrate to improve their socioeconomic status.

#### Migration Experience

Participants discussed their experiences with migration in terms of time in the US and the experience evolved as they spent more years in the country. Most came to the US without migratory documents, but many were able to normalize their status with time. The main problems highlighted by the participants were feeling discriminated, judged or rejected by the new environment, challenges learning English, and lack of documentation which, in some cases, resulted in abuse by policemen, and feelings of fear, loneliness, and difficulty adapting to a new country. Participants said these situations directly impacted their mental health.

#### Physical and Mental Health

Participants expressed that they did not feel that their physical health had been affected by living in the US. However, they considered that living in the US had negatively impacted their mental health. They again identified being undocumented, as well as long work days, having left family behind in Mexico, language barriers, and feeling unable to adapt to the new country as factors affecting their mental health. They expressed that these situations left them feeling frustration, sadness, anxiety, stress, fear, depression, desperation, and like they do not belong in either Mexico or the US. Some women also mentioned problems in the current relationship with their partners as a factor affecting their mental health, which they said generated emotional distress and poor interactions with their social support networks.

### Implementation and Process Evaluation

The ten virtual sessions were implemented as planned and had an average duration of 50 min. After the first in-person session in February 2020, participants had difficulty traveling to the Consulate and all sessions were then conducted virtually.

The two process evaluations yielded perfect satisfaction scores with all participants strongly agreeing that they were satisfied with the helpfulness of the topics, quality and clarity of the materials, the virtual platform, and the skills of the team psychologist conducting the sessions (using clear language, being respectful, etc.). There was 10% dissatisfaction with two sessions due to excessive duration (data not shown).

### Pre- and Post-evaluation

#### Quantitative Results

Participants significantly increased their scores on knowledge of protective factors and coping skills, and had lower scores in the Kessler Scale of Psychological Distress (all *p* < 0.05) (Table 2).

**Table 2:** Pre- and post-evaluation scores for protective factors and coping mechanisms knowledge, and psychological distress among Mexican immigrants who participated in a mental health promotion intervention (*n* = 9).

	**Intent to treat (*****n*** **=** **25)**			**Complete case (*****n*** **=** **9)**^b^		
**Scales [median (inter-quartile range)]**	**Pre-**	**Post-**	***p*-value^a^**	**Pre-**	**Post-**	***p*-value^a^**
Protective factors knowledge	111 (100, 120)	115 100, 124)	0.02	114 (108, 130)	137 (120, 140)	0.01
Coping mechanisms knowledge	96 (88, 108)	99 (90, 110)	0.03	96 (85, 104)	100 (90, 125)	<0.01
Kessler scale of psychological distress	3 (2, 3)	2 (2, 3)	0.01	3 (2, 4)	2 (1, 2)	0.02

a *Wilcoxon signed-rank test for non-parametric data*.

b *Participants who attended <70% of the sessions or did not complete the post-evaluation were excluded*.

#### Qualitative Results

Based on the theoretical framework, the intervention curriculum and the pre- and post-semi-structured interviews, the themes identified in the qualitative analysis were social support networks, adaptability, positive mental health habits, emotional regulation, stress management when facing a problem, and the role of the intervention during the COVID-19 pandemic. Many participants reported progress in the areas covered by the intervention and described using some of the techniques that were recommended during the sessions. They also identified some challenges applying the new knowledge and reported that they continue to work on those areas (Table 3).

**Table 3:** Qualitative results for pre- post- evaluation of a mental health promotion intervention for Mexican immigrants.

**Theme**	**Description**	**Sample quote**
**Social support networks**		
Pre- evaluation	Participants described having networks such as family members (partners, parents, siblings) and identified priests as a secondary source of social support. Many mentioned that their networks are small, limited to family, almost none mentioned having large friend networks. They mentioned that they spend time together with family on a daily basis but seldom with friends. Strategies to broaden their social networks were incorporated into the sessions.	“*Mmm… I have few friends, don't have too many, in general twice a month, something like that, we do something with family, someone invites me to their home, or I invite them to mine.”* **(Health assistant, US permanent resident, 36 years, pre-interview)**
Post-evaluation	Participants were better able to conceptualize their social support networks, for example people with whom they feel supported in case of a difficult situation or emergency. Almost all participants said that they felt supported by their nuclear family living in Mexico or in the US, such as spouse, children, siblings, and brothers or sisters in law. They also in general identified larger social support networks that included friends, community organizations, coworkers, and mental health professionals or spiritual counselors (including priests). They also related that they were spending time with their nuclear family every day, either in person with those living in the US or via video calls or text messaging with those living in Mexico. They also mentioned that the increased interactions with extended family or friends were through video calls or messages due to the COVID-19 pandemic.	“*With my close family, meaning my parents, sisters, with…After the sessions where we talked about how we should broaden the circle of persons who we can trust I have tried to become closer with people I know to try to broaden it.”* **(Bank assistant, DACA recipient, 25 years, post-interview)**
**Adaptability**		
Pre-evaluation	For some participants, adapting to the US was easy because they desired to live in this country or because they migrated when they were very young, or because of what they identified as their individual capacity to adapt quickly to new environments. Others expressed that it was difficult due to the radical change of environment, traditions, and language; however, they were eventually able to achieve it.	“*It does affect you to be in another country because imagine that you from one day to another had a job, family, friends and you leave it and well adapting to the language, the traditions, I did not know people, it was very difficult for me at the beginning to get used to it and to help my children at school, it can be quite frustrating.”* **(Housewife, US Citizen, 54 years, pre-interview)**
Post-evaluation	Participants expressed that they felt adapted and willing to adapt to new situations.	“*I feel adapted, but I would also like to go back to my country, things are what they are, if it is not possible that is what it is and that is it, right?”* **(Supervisor, DACA recipient, 36 years, post- interview)**
**Positive mental health habits**		
Pre-evaluation	Practices which participants used were using music or relaxation videos, drinking tea, or using relaxing oils, excercising, or putting their trust in a superior being. A few participants said that they did not take any actions to care for their mental health.	“*Well, I don't know, I like when I have time to listen to relaxing music, to help me relax the mind and all that. But, well, is the only thing that I do, try to take teas to not be stressed, or something like that. But really, well I do not think I take care of my mental health. And well, like I said I try not to.. not to pay too much attention to stuff and that has really helped me.”* **(Supervisor, DACA recipient, 36 years, pre- interview)**
Post-evaluation	Participants described that now they understood better the importance of taking care of their mental health; also, that they were practicing the techniques that they learned more frequently, such as diaphragmatic breathing, full breathing, relaxing activities, stop thinking about work worries when they are not working, and regulating their emotions of fear and anxiety, not putting pressure on themselves, excercising, sleeping at least 8 hours per day, eating healthy, engaging in hobbies, taking the sun, and positive thinking. A couple of participants mentioned difficulties with healthy eating and not engaging in any activity.	“*I have done many of the exercises that we covered in the workshops like the full breathing or that one that… How do you call it? Diaphragmatic I think, that one where you fill and empty the stomach, I have tried to let go of my thoughts and I go on walks to take care of my mind.”* **(Legal assistant, US citizen, 37 years, post-interview)**
**Emotion regulation**		
Pre-evaluation	The main emotions identified were anger, fear and sadness. Participants in general mentioned that they tried to avoid feeling or confronting these emotions but identified them as being constantly present in their daily lives.	“*Mmmm… when I get angry, I am often in my temperament very easy to anger and yell a lot, I get pretty exalted.”* **(Health assistant, permanent resident, 36 years, pre-interview)** “*The hardest is being without papers, because well my mom can come to visit us, to say that I miss my mom, well no, thanks God she can come to see us, the hardest is that being without papers, that is what worries one, what is going to happen with you. I live with that fear.”* **(Housewife, undocumented, 39 years, pre interview)**
		“*I get sad and I go upstairs to my room to pray or something, or sometimes I even cry.”* **(Housewife, US citizen, 54 years, pre-interview)**
Post-evaluation	Anger: they described dealing with anger in healthier ways such as thinking about the consequences before acting, breathing, and using motion release. A participant said that they are still working on dealing with their anger and that they still show it in an explosive way. Another said that they do not get angry. Fear: they described a decrease in the emotion of fear or dread, the most common response was that they had not felt it lately. A few participants did feel it especially related with the COVID pandemic or because of thoughts that something bad was going to happen. They used some of the emotion regulation techniques such as stopping negative thoughts and exchanging them for positive or facing their fears to control them. There was also a participant that identified that fear detonates other emotions such as anxiety or hopelessness. Sadness: related to sadness, participants said that when they felt it they had used one of the strategies learned in the workshops, such as seeking their social support network to talk about it, and this helped them, also they thought about something else or prayed as a way to control it. A minority said that they had not felt it and did not use any strategy.	“*When I get angry, I have tried to stop yelling a lot, sometimes I remember and I continue to work on that.”* **(Health assistant, permanent resident, 36 years, post-interview)** “*I try with fear, as well as not letting it affect me so much thinking about a situation that I don't know if it will happen, trying not to be thinking and thinking that it hasn't arrived yet. If solutions can be found to solve the problem that is causing fear.”* **(Bank assistant, DACA recipient, 25 years, post-interview)** “*In the sadness, well, sometimes, well, I cry and it helps or I talk, talk to the person, my son or my husband and I explain that it was not right and after that one can express themself better, and well try to talk about it and sort it out.”* **(Housewife, US Citizen, 54 years, post- interview)**
**Capacity to manage stress when facing a problem**		
Pre-evaluation	Participants were not able to define what stress means, however they did acknowledge living situations and symptoms related to it.	“*Mmmm… It is when someone has something to do things that is stress but when it is excessive that is distress and that is the one that hurts you.”* **(Housewife, 37 years, undocumented, pre interview)**
Post-evaluation	Participants defined the concept of stress and some signs and symptoms such as headache, hair loss, neck pain, tiredness and psychological symptoms such as tension and mental fatigue. To manage their stress they described having used one or many of the techniques covered in the sessions, such as meditation, mindfulness, directed fantasy, full and diaphragmatic breathing, Jacobson progressive relaxation technique, technique to stop thinking, and exercise.	“*Well I think that the same things that I have done have helped me, I do 30 breathings three times and that I only did once. I do breathing in the morning, laying down when I wake up, but turns out I fell asleep ha,ha,ha, then I did other exercises. What I still cannot do correctly is the breathing when you should think of nothing, thinking about nothing, I cannot, many things come to mind all the time and sometimes I think silly things. Well, that is how I am doing my breathing and something comes to mind and I say, well what am I doing, and then I concentrate again in the breathing and I think I cannot keep it for long, ha,ha.”* **(Housewife, undocumented, 56 years, post interview)**
**COVID-19**		
Pre-evaluation	The basal evaluation was conducted before the COVID-19 pandemic reached the United States. The theme did not appear during the pre-implementation evaluation. The COVID-19 pandemic was an unexpected event for the participants. Predominant emotions emerged in the participants who were planned to address during the educational sessions. However, not for the context of pandemic and confinement that the world was experiencing. Some participants were coping many days of confinement due to quarantine, exposure to the media, social networks with both trustworthy and false information that only instilled fear, others were developing essential jobs. All these experiences caused them to experience fear, anxiety, stress, and worry of contracting the disease or that it was contracted by a member of their family, children or husband.	“*And with fear because I try to think positive, if I was afraid of the virus [coronavirus] for my girl, because if something happens to her or to me, I can't imagine leaving her alone, but I've taken care of myself, we do everything to clean and we don't go out.”* **(Legal assistant, US citizen, 37 years, post-interview)**
Post-evaluation	The COVID-19 pandemic was an event that exposed to the participants to an unprecedented situation, triggering negative emotions. However, some participants stated using at that time some of the tools learned in the educational sessions to coping these negative emotions and they were useful.	“*With much more confidence, with many more tools to reach out and cope the current situation of the coronavirus, because I believe all this came at the right time, because who would have known that all this was going to happen and at the same time that you were teaching us. The pandemic showed up and it has affected everyone, there is a lot of fear among people and all the things that are put on social media that are lies and many are true.”* **(Housewife, US Citizen, 54 years, post- interview)**

## Discussion

Despite their large numbers and high levels of emotional distress and mental health problems, reaching the Mexican immigrant population through mental health interventions has been very challenging. Little research has addressed this problem. We leveraged the reach and access of the VDS Atlanta to design and pilot a mental health promotion intervention for Mexican immigrants that was successful in improving psychological distress and knowledge among participants who attended at least 70% of the sessions. These results are encouraging and suggest that efforts to support protective mental health factors and teach coping strategies in groups has the potential to improve mental health outcomes in a distressed population.

This is the first study reporting on a mental health promotion intervention for Mexican immigrants implemented through a VDS; however, other similar preventive interventions have been conducted with ethnic and racial minority groups in the US. Most have focused on mental health assessments and referrals (12). Raval et al. (13) conducted a mental health promotion intervention and were successful improving internalizing and externalizing behaviors among minority youth in New York City. Blumenthal et al. used community based participatory research to develop mental health promotion interventions for African Americans and Latinos that resulted in better self-reported physical health and a decrease in depression symptoms (14). Another commonly-used approach among community-based mental health initiatives has been through *promotoras* (12). The *Promotoras* Model, where community health workers receive basic training to provide health education or promotion in their communities, has been used successfully in many areas of health and is increasingly used for group or individual mental health promotion interventions. The ALMA (*Amigas Latinas Motivando el Alma*/Latina Friends Motivating the Soul) pilot intervention used this model for a peer- support and education intervention was successful improving depression symptoms, acculturative stress and coping mechanisms among Latina immigrants in North Carolina (15). This is consistent with our findings and highlights the potential impact of community health promotion interventions to improve the health of Latino immigrants in the US. Further, the fact that our study is imbedded into an ongoing, national project that already conducts some screening and referrals could contribute to scaling up improved versions of this intervention.

An important finding of this study was high levels of attrition. High attrition and underrepresentation of Latinos and other racial and ethnic minorities have been important challenges in health research (31). In this study, through the VDS, we were able to reach and enroll a sample of Mexican immigrants, a population that based on the various societal, cultural and economic challenges has been difficult to include in health interventions, but several factors limited the retention of more than half of the sample. The main impediment for participants to join the sessions was problems with technology or signing into the virtual platform. In this sense, technological advances provide an opportunity to deliver health interventions, including in the mental health field, however, a digital divide has been identified where minoritized populations tend to have less access to technological resources (32). If this divide is not addressed, technology could exacerbate disparities in health promotion and care, which is a particular concern after the COVID-19 pandemic increased the need for remote and online interventions (33). A lesson-learned from this pilot-intervention was the need to address technological barriers before the start of the program. Additionally, some participants reported dissatisfaction with session length, and it is possible that 10 session was excessive; future program iterations should assess the feasibility and impact of delivering the program in shorter and fewer sessions.

Fear and anxiety were reported by participants both as barriers to attend the sessions and as one of the main emotions affecting their mental wellbeing. Participants identified being undocumented, working conditions, distance from family, language barriers, and adaptation challenges as factors leading to these emotions. This is consistent with other studies that have identified migration and socio-cultural factors as specific barriers affecting the mental health of Latino immigrants in the US (34). Others have also identified fear and anxiety as factors related to attrition (29), highlighting the importance of addressing these factors as part of health interventions, especially those targeting immigrants and racial or ethnic minorities; this could potentially improve retention and translate into better mental health outcomes (35). Further research is needed to identify potential approaches to address these factors and increase the reach of mental health promotion interventions in this population.

The small sample size was another limitation of this study besides attrition; however, we were still able to detect differences in the sample that completed the final evaluation, supporting the efficacy of the intervention. The COVID-19 pandemic was a challenge that made it even harder for some participants to attend the sessions, but the importance of having this form of support during the beginning of the pandemic was highlighted by participants in the final evaluation. Strengths of this study include the formative research that allowed us to design a culturally-relevant intervention and to take advantage of the community networks and programs of the VDS; the use of theoretical frameworks that incorporate protective factors, coping skills and emotional education for mental health promotion in marginalized populations; and the mixed methods evaluation that allowed us to assess the impact and also understand the psychosocial factors around attrition and effectiveness.

In conclusion, we developed and implemented a mental health promotion intervention for Mexican Immigrants through the VDS program in Atlanta that resulted in increased knowledge and lower distress among those who attended at least 70% of the sessions. The impact of this results was attenuated by high attrition that resulted from technological challenges and psychosocial barriers associated with migration and other socio-economic factors. In order to achieve health equity, particularly in mental health, it is important to continue to implement and study culturally relevant mental health promotion interventions for Latino immigrants and other racial and ethnic minorities that address the particular challenges faced by these populations.

## Data Availability Statement

The datasets presented in this article are not readily available because of the small number of participants and the sensitive nature of this data. Requests to access the datasets should be directed to IG, inegonza@iu.edu.

## Ethics Statement

The studies involving human participants were reviewed and approved by National Institute of Public Health Ethics Review Board. The patients/participants provided their written informed consent to participate in this study.

## Author Contributions

DM, TG, MM, and IG contributed to conception and design of the study. DM conducted the data collection, implemented the pilot intervention, and analyzed the data. TG, MM, and IG guided the analysis of the data. MG and AF contributed to the interpretation of the data and wrote sections of the manuscript. DM and IG wrote the first draft of the manuscript. All authors contributed to manuscript revision, read, and approved the submitted version.

## Funding

The Ventanilla de Salud Atlanta at the time of the study was funded through a shared cost agreement between Emory University and Mexico's Secretary of Health (SSA) and Institute for the Mexicans in the Exterior (IME). The intervention was funded through funds that were destined for mental health promotion by SSA and IME in 2019. IG received funds from the National Heart, Lung and Blood Institute as part of UCSF's Research in Implementation Science for Equity small research project (5R25HL126146).

## Conflict of Interest

IG served as Emory University's director of the Ventanilla de Salud Atlanta program at the time of the implementation of the study. The remaining authors declare that the research was conducted in the absence of any commercial or financial relationships that could be construed as a potential conflict of interest.

## Publisher's Note

All claims expressed in this article are solely those of the authors and do not necessarily represent those of their affiliated organizations, or those of the publisher, the editors and the reviewers. Any product that may be evaluated in this article, or claim that may be made by its manufacturer, is not guaranteed or endorsed by the publisher.
